# Thyroid Lobectomy for Low-Risk 1–4 CM Papillary Thyroid Cancer is not Associated with Increased Recurrence Rates in the Dutch Population with a Restricted Diagnostic Work-Up

**DOI:** 10.1007/s00268-022-06813-5

**Published:** 2022-10-27

**Authors:** J. F. Lin, P. M. Rodriguez Schaap, M. J. H. Metman, E. J. M. Nieveen van Dijkum, C. Dickhoff, T. P. Links, S. Kruijff, A. F. Engelsman

**Affiliations:** 1 Department of Surgical Oncology, University Medical Center Groningen, University of Groningen, P.O. 30.001, 9700 RB Groningen, The Netherlands; 2 Department of Surgery, Location VUmc Cancer Centre Amsterdam, Amsterdam University Medical Centre, Postbus 7057, 1007 MB Amsterdam, The Netherlands; 3 Division of Endocrinology, Department of Internal Medicine, University Medical Center Groningen, University of Groningen, Groningen, The Netherlands

## Abstract

**Introduction:**

The 2015 American Thyroid Association guidelines recommend to de-escalate treatment such as Thyroid lobectomy instead of total thyroidectomy for 1–4 cm papillary thyroid cancer (PTC). Dutch guidelines endorse restricted work-up for thyroid incidentalomas recommending only fine needle aspiration in case of a ‘palpable thyroid nodule’. This diagnostic work-up algorithm may result in the identification of less indolent PTCs and may lead to a patient population with relatively more aggressive PTCs. This study aims to retrospectively analyze recurrence rates of low-risk 1–4 cm PTC in the Netherlands.

**Methods:**

From the national cancer registry, patients with low-risk 1–4 cm PTC between 2005 and 2015 were included for analysis. Disease free survival (DFS) and overall survival were compared between patients who underwent TT ± RAI and TL without RAI. Post-hoc propensity score analysis was performed correcting for age, sex, T-stage, and N-stage.

**Results:**

In total 901 patients were included, of which 711 (78.9%) were females, with a median follow-up of 7.7 years. TT was performed in 893 (94.8%) patients. Recurrence occurred in 23 (2.6%) patients. Multivariable analysis showed no significant correlation between extent of surgery and DFS (*p* = 0.978), or overall survival (*p* = 0.590). After propensity score matching, multivariable analysis showed no significant difference on extent of surgery and recurrence.

**Conclusion:**

Low-risk PTC patients with 1–4 cm tumor who underwent TL showed similar recurrence rates as those who underwent TT ± adjuvant RAI, which suggests that TL can be sufficient in treating low-risk 1–4 cm PTC, possibly reducing morbidity of these patients in the Netherlands.

## Introduction

The incidence of thyroid cancer has tripled in the past decades and now represents 2.1% of all new cancer cases worldwide [1]. Papillary thyroid cancer (PTC) is the most common histological subtype and accounts for more than 85% of all thyroid malignancies [2]. Treatment of PTC involves surgery, usually followed by radioactive iodine ablation (RAI), resulting in 15-year overall survival rates as high as 95% [1]. Based on findings from U.S. and Japanese population studies, TL for low-risk 1–4 cm PTC results in comparable overall survival, and less morbidity such as hypoparathyroidism, hematoma and laryngeal nerve injury when compared to TT ± RAI [3]. Consequently, the American Thyroid Association (ATA) guidelines recommend a less aggressive approach for low-risk 1–4 cm PTC, i.e. thyroid lobectomy (LT) instead of total thyroidectomy (TT) followed by RAI [4–7].

In contrast to the ATA guidelines, the 2015 Dutch thyroid cancer guidelines still recommend TT ± RAI for both low- and high-risk PTC larger than 1 cm. However, in contrast to the ATA guidelines, the Dutch guidelines recommend more restricted work-up for thyroid incidentalomas recommending only fine needle aspiration (FNA) in case of a ‘palpable thyroid nodule’. [8, 9] Multiple studies showed that increased use of imaging modalities leads to increased number of PTC diagnoses, mostly without clinical relevance [10, 11]. Subsequently, using a restrictive diagnostic work-up algorithm may result in the identification of less PTCs with an indolent nature and may lead to a patient population with relatively more aggressive PTCs. Therefore, 2015 ATA guidelines recommendations to de-escalate treatment for low-risk 1–4 cm PTC may be less applicable to the Dutch population [9, 12].

To assess oncological safety of the 2015 ATA guideline recommendations in the clinical practice of a restricted diagnostic work-up in the Netherlands, we aimed to analyze the overall survival and risk of recurrence of low-risk 1–4 cm PTC.

## Material and methods

### Data collection

Data was obtained from the national database of the Netherlands Comprehensive Cancer Organization (IKNL) and the national network and registry of histo- and cytopathology in the Netherlands (PALGA) from 2005 to 2015. Patients were identified by means of their pathology report and were reclassified according to the 8th edition of the AJCC TNM staging. Patients older than 18 years with cT1-cT3 papillary thyroid cancer were identified from the IKNL database and were linked with the PALGA database through de-identified patient numbers. Pathology reports were reviewed, and clinical data were collected (age at diagnosis, sex, initial surgical treatment, vital status, pathology and RAI treatment). Only patients with low-risk PTC were included for analysis, which was defined as clinically node negative (cN0) or ≤ 5 pN1a positive nodes, unifocal disease, and no microscopic extrathyroidal extension, venous invasion or lateral lymph node metastasis (pN1b) at initial therapy. Patients were excluded in case of PTC of unknown size or PTC ≤ 1 cm and > 4.0 cm, surgery other than TL or TT, non-invasive follicular thyroid neoplasm with papillary-like nuclear features (NIFTP), positive extrathyroidal extension, and distant metastasis at diagnosis on pathology reports were excluded. This study was approved by the Amsterdam UMC medical ethical committee (study number 2020.449).

### Follow-up data on recurrence and overall survival

Follow-up data was obtained up to December 2019. The IKNL verified survival status of patients through the Personal Records Database (BRP), which contains personal data of all people who live in the Netherlands. Overall survival was defined as time till death from any cause. Using the PALGA database, recurrence was defined as histology or cytology proven locoregional recurrence or distant metastases after a documented disease-free period of at least 12 months after initial surgical treatment. Disease free survival was measured as time after primary treatment until recurrent disease.

### Statistical analysis

Statistical analysis was performed using IBM SPSS (version 25.0.0.0. SPSS Inc., Chicago) and GraphPad Prism (version 8.0, GraphPad Software Inc., San Diego). Descriptive data are presented as mean with standard deviation (SD), median with interquartile range (IQR), or as a percentage (%). Continuous variables were analyzed by Mann–Whitney’s U-test for non-normal distributed data or Student’s t-test for normally distributed data. Categorical variables were compared using the Chi-Square test, and one-way ANOVA to compare means of continuous variables. Both overall survival and disease-free survival (DFS) were estimated by Kaplan-Meijer curves and log-rank test. The Cox proportional hazard model was performed to identify factors significantly associated with disease recurrence and overall survival. To reduce bias due to confounding variables, post-hoc propensity score matching for age, sex, T-stage, and N-stage was performed with a ratio of 1:2 (TL: TT, respectively). Statistical significance was considered at p < 0.05.

## Results

### Patient inclusion and characteristics

**Fig. 1:**
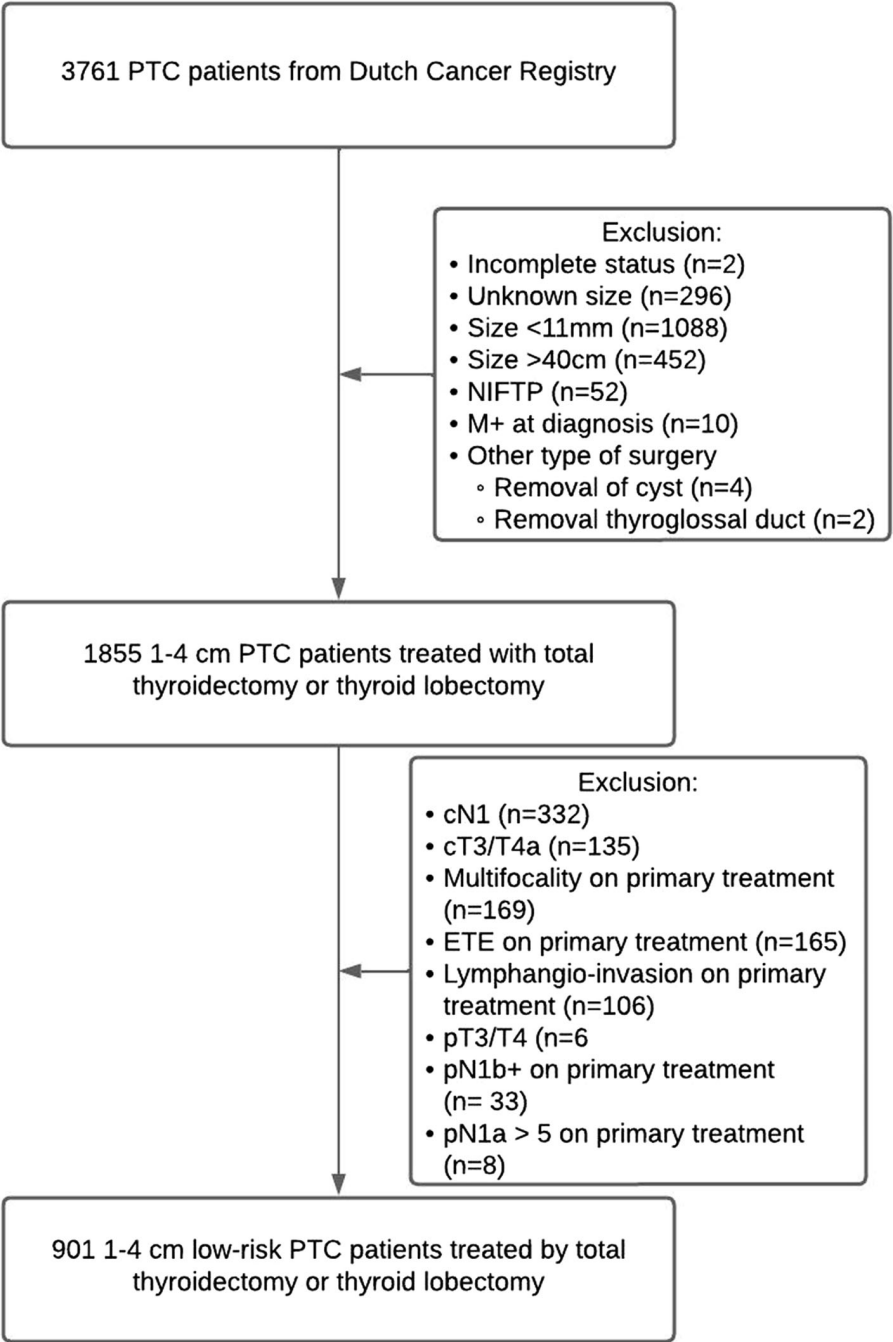
Flowchart of patient inclusion and exclusion criteria. Abbreviations: PTC, papillary thyroid cancer; NIFTP, non-invasive follicular thyroid neoplasm with papilary like nuclear features; ETE, extrathyroidal extension

Between 2005 and 2015, a total of 3761 patients diagnosed with PTC were surgically treated in the Netherlands. Of these, 2860 patients did not meet inclusion criteria and were excluded from analysis (Fig. 1). In total, 901 patients with low-risk 1–4 cm PTC were included for analysis of which 711 (78.9%) were female and 190 (21.1%) male. Baseline characteristics are described in Table 1. The mean age at diagnosis was 47.3 years (SD ± 15.1). Patients undergoing TT had a more advanced T-stage when compared to patients undergoing TL (p = 0.003). Subsequently, adjuvant RAI was given to 756 (88.7%) of all TT patients.

**Table 1:** Baseline characteristics and outcomes according to surgical treatment for low-risk 1–4 cm PTC

	Total (*n* = 901)	TL (*n* = 49, 5.2%)	TT (*n* = 893, 94.8%)	*p*-value
Age (years, mean ± SD)	47.3 ± 15.1	51.1 ± 16.2	47.0 ± 15.0	0.063
**Sex**				
Men (%)	190 (21.1%)	10 (20.4%)	180 (21.1%)	0.804
Women (%)	711 (78.9%)	39 (79.6%)	672 (78.9%)	
Follow-up median time (years, IQR)	7.7 [IQR; 5.7–10.6]	7.5 [IQR; 5.3–11.6]	7.8 [IQR; 5.7–10.6]	0.312
**pT-stage (%)**				
pT1b	475 (52.7%)	36 (73.5%)	439(51.5%)	**0.003**
pT2	426 (47.3%)	13 (26.5%)	413 (48.5%)	
**pN-stage (%)**				
pNx	626 (69.5%)	37 (75.5%)	589 (69.1%)	0.550
pN0	193 (20.6%)	10 (20.4%)	183 (21.5%)	
pN1a (≤ 5 nodes)	82 (9.1%)	2 (4.1%)	80 (9.4%)	
RAI (%)	756 (83.9%)	0	756 (88.7%)	** < 0.001**
Recurrence	23 (2.6%)	0	23 (2.7%)	0.244

*p*-value < 0.05 is considerd statistically significant

*TL* thyroid lobectomy; *TT* total thyroidectomy; *SD* standard deviation; *IQR* interquartile range; *RAI* radioactive iodine therapy

### Risk of recurrence

**Fig. 2:**
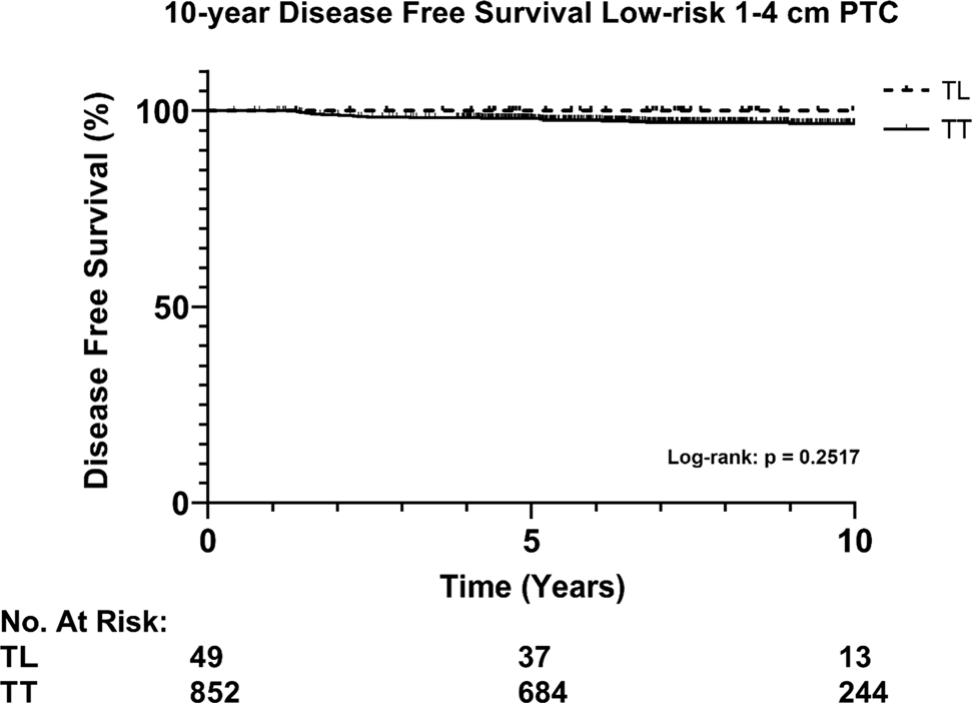
Disease free survival of low-risk 1–4 cm PTC stratified by the extent of surgery. Abbreviations: PTC, papillary thyroid cancer; TL, thyroid lobectomy; TT, total thyroidectomy

**Fig. 3:**
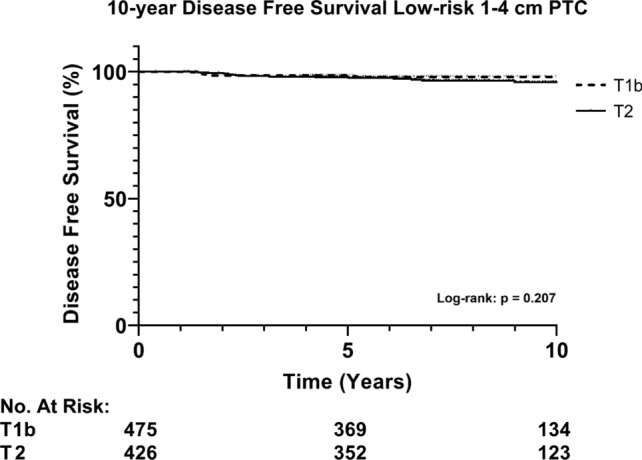
Disease free survival of low-risk 1–4 cm PTC stratified T-stage (pT1b versus pT2). Abbreviations: PTC, papillary thyroid cancer

Overall, recurrence occurred in 23 (2.6%) patients during a median follow-up of 7.7 years [IQR; 5.7–10.6]. Subsequently, recurrences only occurred in the TT group and presented as localized disease. No statistical difference in recurrence rate was found between patients undergoing TL versus TT ± RAI (0% vs. 2.7%, *p* = 0.244). Additionally, Kaplan Meier analysis for recurrence showed no difference in 10-year disease free survival (DFS) for TL versus TT ± RAI (respectively, 100% and 96.8%, *p* = 0.252, Fig. 2). Also, no significant difference in 10-year disease free survival was found when stratified by T-stage (pT1b vs. pT2), (Fig. 3, *p* = 0.207). Furthermore, multivariable analysis of low-risk 1–4 cm PTC accounting for sex, age, T-N-status and extent of surgery (TL vs. TT) showed that only N-status (pN1a with ≤ 5nodes) was an independent risk factor for risk for recurrence (HR 4.969, CI [2.015–12.253], *p* < 0.001, Table 2). Sex, age > 55 years, T-stage and extent of surgery were not significantly correlated with the risk for recurrence.

**Table 2:** Multivariable cox regression for recurrence hazard with extent of surgery for low-risk 1–4 cm PTC

Risk factor	Hazard ratio [95% CI]	*p*-value
Sex (men)	–	0.126
Age (> 55)	–	0.562
Extent of surgery (TL)	–	0.978
**pT-stage**		
pT1b	Referent	
pT2	–	0.200
**pN-stage**		
pNx/N0	Referent	
pN1a (≤ 5 nodes)	4.97 [2.015 – 12.253]	** < 0.001**

*p*-value < 0.05 is considerd statistically significant

*TL* thyroid lobectomy; *CI* confidence interval

### Overall survival

**Fig. 4:**
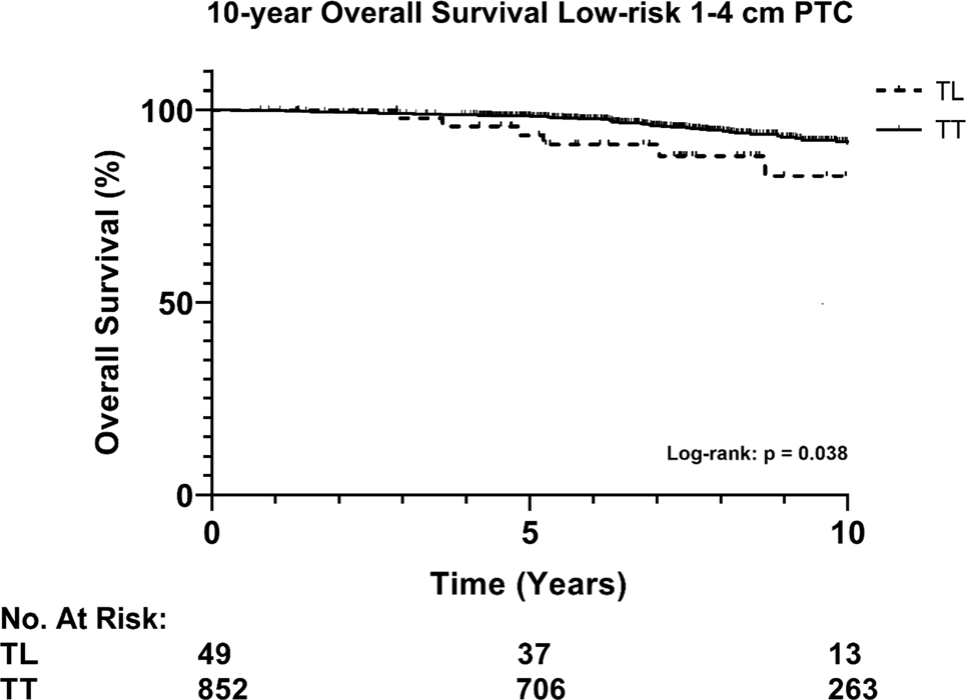
Overall survival of low-risk 1–4 cm PTC stratified by the extent of surgery. Abbreviations: PTC, papillary thyroid cancer; TL, thyroid lobectomy; TT, total thyroidectomy

**Fig. 5:**
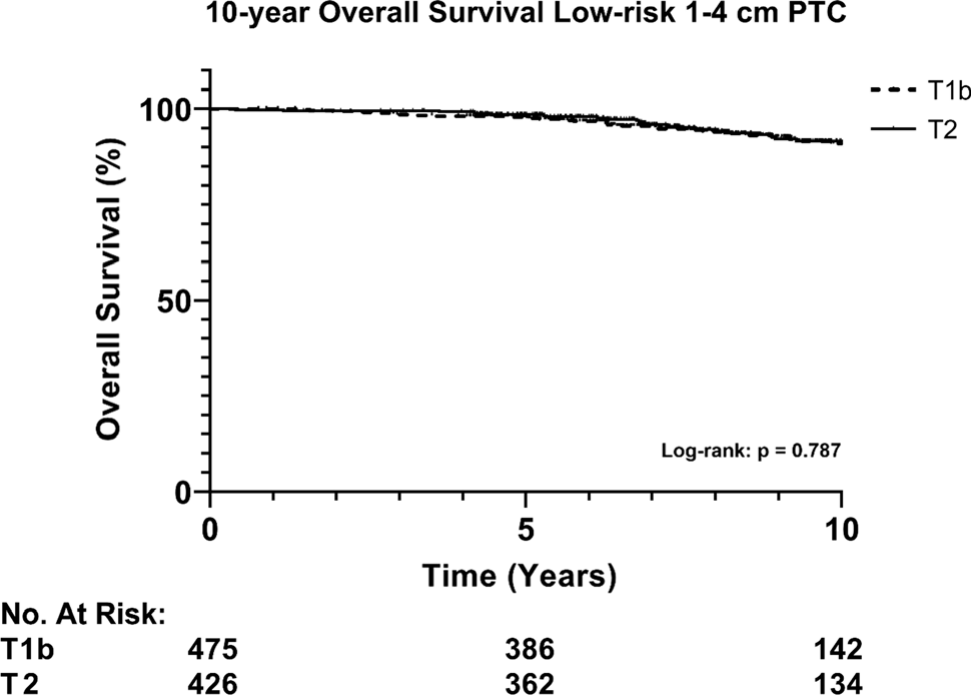
Overall survival of 1–4 cm PTC stratified by T-stage (pT1b versus pT2). Abbreviations: PTC, papillary thyroid cancer

Kaplan Meier analysis for overall survival in patients with low-risk 1–4 cm PTC showed a 10-year overall survival of 82.8% and 91.4% for TL and TT, respectively (*p* = 0.038, Fig. 4). When stratified by size (pT1b vs. pT2), no significant difference in 10-year overall survival is seen (Fig. 5, *p* = 0.787). Cox regression for low-risk PTC 1–4 cm showed that, when accounting for sex, age, and T-N -stage, TL was not an independent risk factor for overall survival (*p* = 0.112). Male sex, age > 55 years, and pN1a (≤ 5 nodes) were all significantly correlated to worse overall survival (Table 3).

**Table 3:** Multivariable cox regression for overall survival with extent of surgery for 1–4 cm low-risk PTC

Risk factor		Hazard ratio [95% CI]	*p*-value
Sex (men)		2.58 [1.60–4.17]	** < 0.001**
Age (> 55)		4.07 [2.483–6.67]	** < 0.001**
Extent of surgery (TL)		–	0.590
**pT-stage**			
pT1b		Referent	
pT2		–	0.682
**pN-stage**			
pNx/N0		Referent	
pN1a (≤ 5 nodes)		2.220 [1.16–4.25]	**0.016**

*p*-value < 0.05 is considerd statistically significant

*TL* thyroid lobectomy; *CI* confidence interval

### Post-hoc propensity score matching

Patient characteristics after propensity score matching are described in appendix Table 4. Multivariable analysis showed no significant difference between extent of surgery and recurrence (appendix Table 5) and overall survival (appendix Table 6). N-stage was not considered a significant risk factor on overall survival.

**Table 4:** Baseline characteristics and outcomes according to surgical treatment for low-risk 1–4 cm PTC

		Total (*n* = 147)	TL (*n* = 49, 33.3%)	TT (*n* = 98, 66.7%)	*p*-value
Age (years, mean ± SD)		51.7 ± 15.6	51.1 ± 16.2	52.0 ± 15.4	0.857
**Sex**					
Men (%)		25 (17.0%)	10 (20.4%)	15 (15.3%)	0.438
Women (%)		122 (83.0%)	39 (79.6%)	83 (84.7%)	
Follow-up median time (years, IQR)		9.3 [IQR; 6.9–12.2]	8.4 [IQR; 5.7–12.5]	9.5 [IQR; 7.6–12.0]	0.220
**pT-stage (%)**					
pT1b		105 (71.4%)	36 (73.5%)	69 (70.4%)	0.699
pT2		42 (28.6%)	13 (26.5%)	29 (29.6%)	
**pN-stage (%)**					
pNx		105 (71.4%)	37 (75.5%)	68 (69.4%)	0.492
pN0		33 (22.4%)	10 (20.4%)	23 (23.5%)	
pN1a (≤ 5 nodes)		9 (6.2%)	2 (4.1%)	7 (7.1%)	
RAI (%)		106 (72.1%)	0	79 (80.6%)	** < 0.001**
Recurrence		5 (3.4%)	0	5 (5.1%)	0.108

*p*-value < 0.05 is considerd statistically significant

*TL* thyroid lobectomy; *TT* total thyroidectomy; *SD* standard deviation; *IQR* interquartile range; *RAI* radioactive iodine therapy

**Table 5:** Multivariable cox regression for recurrence hazard with extent of surgery for low-risk 1–4 cm PTC

Risk factor		Hazard ratio [95% CI]	*p*-value
Sex (men)		–	0.979
Age (> 55)		–	0.986
Extent of surgery (TL)		–	0.965
**pT-stage**			
pT1b		Referent	
pT2		–	0.475
**pN-stage**			
pNx/N0		Referent	
pN1a (≤ 5 nodes)		11.2 [1.0 – 124.6]	**0.049**

*p*-value < 0.05 is considerd statistically significant

*TL* thyroid lobectomy; *CI* confidence interval

**Table 6:** Multivariable cox regression for overall survival with extent of surgery for 1-4 cm low-risk PTC

Risk factor		Hazard ratio [95% CI]	*p*-value
Sex (men)		3.60 [1.34–9.63]	**0.011**
Age (> 55)		4.07 [2.483–6.67]	**0.009**
Extent of surgery (TL)		–	0.889
pT-stage			
pT1b		Referent	
pT2		–	0.783
pN-stage			
pNx/N0		Referent	
pN1a (≤ 5 nodes)		–	0.982

*p*-value < 0.05 is considerd statistically significant

*TL *thyroid lobectomy; *CI* confidence interval

## Discussion

In this nationwide retrospective study, oncological outcome after TL was comparable with TT ± RAI in low-risk 1–4 cm PTC. We found a 10-year recurrence rate of 2.6% in this group of patients with low-risk 1–4 cm PTC as defined according to 2015 ATA guideline criteria. Interestingly, we show that in a setting of a restricted diagnostic work-up for thyroid nodules, TL resulted in no significant difference in risk of recurrence when compared to TT ± RAI.

The recommendations in the 2015 ATA guidelines were based on both Surveillance, Epidemiology, and End Results Program database (SEER) and National Cancer Data Base (NCDB) analyses. Recurrence and survival data published from these databases all show constant survival rates and recurrence rates regardless of extent of surgery [6, 7, 13]. In addition, TL offers less morbidity such as hypoparathyroidism, hematoma and laryngeal nerve injury when compared to TT ± RAI [3]. Contrarily, TT benefits patients with bilateral tumors, allows adjuvant RAI treatment and facilitates the use of Thyroglobulin (Tg) as a tumor marker to detect residual or recurrent disease during follow-up [4].

In the Netherlands a restrictive diagnostic work-up strategy is standard care for thyroid nodules, recommending diagnostic work-up only for palpable nodules, preventing fine needle aspiration (FNA) biopsies of incidentalomas detected by imaging [8]. Since 2013, the number of PET/CT scans performed grew by 6% annually, and together with the exponential increase in use of US imaging, more incidentalomas are found [10, 11, 14]. However, the Dutch guideline only recommend diagnostic FNA for patients with palpable or symptomatic thyroid nodules, which possibly prevents a significant amount of indolent PTC from being diagnosed, resulting in more intensely selected and possibly more aggressive recorded PTCs in The Netherlands when compared to the U.S. population [8, 9]. In other words, most thyroid incidentaloma based PTCs have indolent behavior and will most likely have only little effect on mortality rates [15]. It makes sense to de-escalate treatment, such as has been advocated in the ATA guidelines, in a society with an aggressive diagnostic work up for every single incidentaloma. Earlier, Metman et al. already illustrated this phenomenon comparing Dutch data with the U.S., describing that the Dutch population shows a lower percentage of 1–2 cm PTC and subsequently a higher percentage of 2–4 cm tumors [9]. As 2–4 cm is a prognostic factor for risk of recurrence, we questioned if de-escalating to TL in the Dutch population is suitable [16].

Within our Dutch population of low-risk 1–4 cm PTC, after multivariable adjustment accounting for sex, age, T- and N-stage, there was no DFS disadvantage associated with TL as compared to TT ± RAI. This remained, even after post-hoc propensity score matched correction for age, sex, T-stage, and N-stage. When stratified by tumor size between 1–2 cm and 2–4 cm, no significant differences in DFS or overall survival was found. Studies have shown varying DFS regarding TL vs. TT ± RAI. A recent systematic review from Chan et al. described that low-risk 1–4 cm PTC patients undergoing TL had a significant higher recurrent rate when compared to patients who underwent TT ± RAI. However, some studies included in this review had patients included with high-risk features such as vascular invasion and extrathyroidal extension as well as T3 + patients [17]. A recent meta-analysis from Rodriguez Schaap et al. on low-risk well differentiated T1-2 PTC only, reported similar recurrence rates after TL as compared to patients who were treated with TT ± RAI (pooled recurrence rate of 2.3% and 2.8% respectively, *p* = 0.48). This meta-analysis, however, did include papillary microcarcinomas in the analysis. In the current study, we strictly selected patients according to the 2015 ATA guideline definitions for low-risk 1–4 cm PTC to minimize selection bias [3].

In this study we report that patients undergoing TL have a worse 10-year OS compared to patients undergoing TT. This was striking for the analysis of U.S. and Japanese population studies which showed that TL is associated with excellent survival in properly selected PTC patients and was decisive for the new recommendation in 2015 ATA guidelines [4–7]. However, in our multivariable analyses, overall survival was not significantly affected by the extent of surgery (TL) but age > 55 years reveals to be the strongest prognostic factor for OS. The Netherlands Cancer Registry unfortunately does not register disease-specific mortality and morbidity, and the exact cause of death of the patients is unknown in this study. Moreover, the difference between the extent of surgery might reflect the efforts of the treating physician to opt for less aggressive surgery for older patients, possibly with comorbidities, to offer a better quality of life. As the main outcome for this study is recurrence, and considering same follow-up lengths for TL and TT patient groups, we believe that having comorbidities has no significant impact on the study results. Nevertheless, previous literature shows that stage I PTC has an excellent prognosis, with disease-specific survival of 98–100% [18]. Therefore, we consider that the risk of recurrence offers a better prognostic value to assess whether a TL or TT should be performed.

The results of this study should be addressed with respect to the limitations of our study based on retrospective data analysis the lack of biochemical and mortality data and study biases. Although appropriate care is taken to reduce selection bias, patients were selected for a procedure based on Dutch guidelines, recommending TT ± RAI for PTC > 1 cm. In the Netherlands, almost 95% of the patients with 1–4 cm PTC were treated with TT ± RAI, showing strict adherence to our national guideline. Moreover, the recurrence rates described in this study might be underreported. In the Netherlands, standard follow-up procedure is performed according to (inter)national guidelines using regular Tg and neck ultrasound. However, the Netherlands Cancer Registry does not incorporate disease recurrence. Therefore, we can only report on cytology or histology proven recurrences and not on radiological or biochemical recurrence, as no follow-up data on ultrasound reports nor serum Tg levels were available.

This study shows that TL can be safely implemented for carefully selected low-risk 1–4 cm PTC in countries with restrictive diagnostic work-up strategies, such as the Netherlands. As histopathology is final only after surgical removal of the affected lobe, it is likely that after resection a portion of clinically low-risk patients will be restaged to intermediate or high-risk PTC following classification criteria of the ATA guidelines. These patients require completion thyroidectomy and might be exposed to higher risk of complications when compared to one-staged total thyroidectomy [19]. Therefore, future studies identifying the true proportion of low-risk 1–4 cm PTC patients in the Netherlands is warranted to analyze the impact of completion thyroidectomy in these patients.

## Conclusion

In the Dutch population with a strict diagnostic work-up strategy for thyroid nodules TL for low-risk 1–4 cm PTC patients showed similar recurrence rates as those who underwent TT ± RAI. This suggests that thyroid lobectomy can be sufficient in treating unifocal 1–4 cm PTC without extrathyroidal extension, venous invasion or clinical evidence of nodal metastases, possibly reducing morbidity of low-risk 1–4 cm PTC patients in the Netherlands.

## Appendix

See Tables 4, 5, 6
